# Notes and Comments

**Published:** 1922-02

**Authors:** 


### NOTES AND COMMENTS.

THE underpayment of Medical Officers of Health indicates how little the public really cares about the prevention of disease. The Mayor, Aldermen, Councillors and Burgesses of a town fail, often enough, to appreciate the work of their Health Department, and this may be chiefly for the reason that, when the work is successful, the department appears but little in the public eye. The quiet, steady and laborious work of public health does not often announce itself dramatically; but if each individual Burgess in a town were asked which was the more important, the tram service or his own individual health, he would reply unhesitatingly that the trams might go to blazes so long as he and his children remained free from plague and pestilence. In the "Leeds Evening Post" of January 6 there appeared a list of the salaries paid to certain of that town's officials. The tramways manager receives £2,530 per annum, the electricity manager £1,500, the gas manager £1,500, the city treasurer £1,386, the sewerage engineer £1,122, and the chief constable £1,135. We have no doubt that all these servants of the public merit entirely the salaries paid to them in their high offices; and we should expect to find that the medical officer of Leeds was equally well paid for his specialist services, since, like them, he has spent many years in training for his work, and has passed a long probation in the public service. His yearly payment is, however, only £935, and even an assistant in the Town Clerk's department receives more than that. We venture to think that there is no general practitioner with a panel practice in Leeds who is receiving less than a thousand a year, and since the Medical Officer of Health is supposed to be the chief medical officer in his town the position is obviously ridiculous as well as distressing. Preventive medicine, paid a starvation wage, will soon cease to attract the best brains in the profession.

IT would appear that the great City of Manchester is intending to follow the practice of Leeds. We do not know if Manchester looks to Leeds for guidance and enlightenment in matters of public health policy; but it would seem that what Leeds thinks to-day Manchester will think to-morrow, for they are proposing to advertise for a Medical Officer of Health at the salary of £1,500 a year. We are sorry to see this city, formerly a leader of thought, commerce and enthusiasm, following the example of a provincial town, and attempting to fill a vacancy at a salary which could not attract any of the better and more learned men in the public health world. No doubt there are many junior men, with public health diplomas and some small amount of academic distinction and practical experience, who would be pleased to double or treble their incomes by leaving a junior post to sit in the public health office at Manchester. We are certain that the appointment of such an one to fill this responsible position would not be advantageous to the great fame of the City. Manchester is not a little and insignificant place whose existence can be disregarded. It is a city with a reputation for courage and enlightenment, a reputation that should be cherished in spite of Leeds, and half-forgotten villages. There are perhaps less than a dozen men in England who could adequately follow Dr. James Niven when he retires from his position at Manchester; and we are certain that the salary offered by the City of Manchester will secure none of these.

INFLUENZA is with us again, and appears to be more of the three-day-fever type than of the variety with pulmonary complications; but since the disease is protean in its manifestations, we hear accounts from different places of new diseases or new symptoms of the disease itself. In one place the outbreak of influenza is mainly catarrhal, in another it is gastro-intestinal, in another it simulates German measles or scarlet fever, or something else. The lay Press likes to discover new diseases; but those who are acquainted with the history of medicine will accept these new discoveries with caution. We are

**NOTES AND COMMENTS—(*cont.*).**

able to publish this month a suggestive article, one of the series of "Notable Epidemics," regarding the influenza of 1918-19, which will be of special interest at the present time. Those of our readers who wish for more information regarding this most interesting of all the diseases should read the admirable Report on the Pandemic of Influenza which was issued by the Ministry of Health in 1920, and can be purchased from H.M. Stationery Office or from any bookseller for the sum of ten shillings.

IN our January number (p. 94) we remarked that very few people, except possibly Clerks to Guardians, would not welcome the redistribution among other authorities of our present Poor Law system; we also referred to the admixture of the aged, the diseased, the imbecile and mentally defective, and the girl waiting to give birth to her illegitimate child, as "loathsome and hateful." Our contemporary, the "Poor Law Officers' Journal," is very cross with us. It pounces on these adjectives and accuses us of applying them to the life of the Poor Law institutions as a whole. We venture to suggest that to assert that the ingredients in a pudding are too numerous, though involving doubt of the plenary inspiration of its original composer, does not necessarily imply condemnation of the sweet course as an institution, still less a denial of the skill, integrity and personal charm of the cook.

REBUTTING accusations that we have not made, our contemporary proceeds to quote a luscious description of Christmas festivities in a workhouse, and the hardly typical instance of a leprous mental case, rejected by hospital and asylum authorities and cared for by "the maligned Poor Law"—neither of which has any obvious relevance to the facts of which we complained. Few people, Medical Officers or others, whose duties have taken them into a workhouse can have failed to observe the mental distress of respectable old or sick folk, forced into association with the vicious and the wastrel. The existing Poor Law did not come down, perfect and unimprovable, from any philanthropic Sinai, and the fact that it is, on the whole, humanely and efficiently administered is no proof that it is not in urgent need of revision.

WE publish in this number Part I. of a suggestive article by Dr. R. J. Ewart, the Medical Officer of Health for Barking, on the subject of Tuberculosis, and some biological considerations regarding it. It is not possible accurately to estimate the financial loss caused year by year to England by the ravages of the tubercle bacillus, nor to arrive at a just conception of the misery and suffering attributable to this disease; but it is common knowledge not only that the effects of tuberculosis are widespread, but also that little is known with certainty about the epidemiology of the "white plague." Any contribution to our knowledge, such as this paper by Dr. Ewart, is therefore welcome. Dr. John Brownlee, working for the Medical Research Council, published in 1918 an investigation into the epidemiology of phthisis in Great Britain and Ireland, and his work, which is now well known, was probably the first real attempt to treat this subject on a scientific basis. He came to the conclusion that pulmonary tuberculosis or phthisis was not one disease but a mixture of three—that there was one type of phthisis which attacked young adults, the commonest age at death being 20–25 years; a second type that affected persons at middle age, when death occurred between 45 and 51 years; and a third type of the disease that attacked persons in old age, the commonest age at death being between 55 and 65 years. Dr. Brownlee did not consider that the young adult type of phthisis was affected to any extent by environment; indeed, the disease seemed to be relatively more common in better-class localities than in poorer places. He was of the opinion that a long-drawn-out epidemic of phthisis is passing over these islands, and that this lengthy epidemic was now on the wane, since the maximum of the young adult mortality from phthisis was somewhere about the middle of last century, and the epidemic of phthisis among the middle-aged had its maximum fully one hundred years ago. Tuberculosis may indeed be an epidemic which is slowly abating; but if this is the case, it has already been many centuries in preparing for its disappearance, since it was well known to the ancient physicians and even to Hippocrates, the father of medicine. Sir Thomas Browne, writing about the time of the Restoration, says in his letter to a friend: "Some think there were few Consumptions in the old world, when men lived much upon milk; and that the ancient inhabitants of this Island were less troubled with coughs when they went naked, and slept in caves and woods, than men now in chambers and feather beds." We commend these words to the consideration of the various epidemiologists of to-day, to those who disbelieve in the influence of environment, in the infectivity of milk and in the protection against tuberculosis afforded by the "Fat-soluble A" Vitamin.

THE Commissioners of the General Board of Control for Scotland comment in their report upon the Stirling District Asylum upon the deaths of eight patients from pulmonary tuberculosis, recorded by Dr. Robert Campbell, the medical superintendent. Six of these, however, had been admitted in the advanced stage of the disease, and were treated in the asylum's sanatorium. The Commissioners urge the importance of these sanatoria in asylums, and remark that previous to their erection the death roll in asylums was four times as great as that from tuberculosis in general hospitals. Sanatoria not only segregate tuberculous patients, but, the Commissioners affirm, educate those addicted to dirty habits. But it is not explained why one part of an asylum should be more educative than another, since insanity and personal uncleanness are familiar companions. The Commissioners can hardly mean that the insane who suffer also from tuberculosis are more addicted to dirty habits than those afflicted only with mental disease. The Board of Control exists to visit and report upon asylums, but in the normal course there is not much to report. It is

**NOTES AND COMMENTS**—(*cont.*).

one of the ironies of institutional management that the asylum without a history has yet to provide its official commentator.

THE new Local Committees formed under the Voluntary Hospitals Commission are getting to work, and the terms and conditions laid down by it for the grants are worth renewed attention. To meet the deficiencies existing on December 31, the basis of computation will be the excess of ordinary cost of maintenance over the income available for meeting it. To enable a hospital to maintain its normal number of beds, the Commission may make emergency grants where it is satisfied that reasonable steps are being, or have been, taken to reduce expense. Where such emergency grants have been made, the balance will proceed *pro rata* against fresh money from other sources, but the total, including emergency grants, must be limited to the sum of £500,000 and is dependent on an equivalent sum of new money being raised. Where the Commission in any area recommends a combined appeal, the aggregate income compared with that of the preceding year of all the hospitals in the area may be the basis of calculation, when the group will be treated as a single institution. The allotment of such a grant, however, will be left at the discretion of the Commission. Where separate hospitals or a group are dealt with, the grant will not exceed half the deficiency existing on December 31. All grants are to be applied wholly to maintenance and not to capital expenditure. Finally, grants will not be paid to any hospital which does not keep its accounts upon the uniform system or such standard form of accounts as the Commission may require; and no hospital will receive a grant which declines to adopt the recommendations of its Local Committee. By these safeguards it is hoped to improve the revenue of the hospitals and also the organisation and economy of their administration. Can any impartial observer affirm that these conditions are unduly onerous or unlikely to encourage co-ordination and fresh voluntary support?

THE precise relation between the closed wards at the London Hospital and the vacant beds at Whitechapel Infirmary has long been a puzzle to the outside public, and articles have appeared in "The Times" on this matter. These brought Mr. E. W. Morris on the scene with the real explanation. He stated that the original suggestion for accommodation between the London Hospital and the Whitechapel Infirmary came from himself, and that, to meet a number of emergency cases, he asked if they might temporarily be sent to the infirmary, so that the waiting cases might not further be delayed. The hospital staff agreed to visit the infirmary; the beds they were to visit were to be called the London Hospital Annexe; the Ministry of Health was invited to approve, and did so; and had no more part nor lot in the matter. Unfortunately, there were, we believe, financial difficulties, though Mr. Morris records the friendly spirit of the Whitechapel Guardians.

IN the New Year's Honours List we saw with pleasure the name of Dr. George S. Buchanan, upon whom a knighthood was conferred by His Majesty. Dr. Buchanan has done solid departmental work for many years both at the Local Government Board and also at the Ministry of Health, of which latter he was the senior medical officer, and more recently he has done special service to this country in international health affairs. We congratulate him upon his well-deserved reward, more especially as distinctions of this nature fall but scantily upon those who are engaged in safeguarding our health. Apart from doctors in government departments, we believe that we are right in stating that a knighthood has been conferred upon only one medical officer of health, namely, Sir Shirley Murphy, who formerly was the medical officer to the London County Council; and, except in his instance, no other medical officer of health has been thus honoured. It is, however, true that during the war several medical officers of health received the intended distinction of the O.B.E., and several more narrowly escaped it. To those from whom came the recommendation for honours we would suggest that there are several big cities and counties in this country which, in many instances, have been served long and faithfully by their medical officers of health who are specialists in the art of preventing disease. Other medical men who are specialists in the use of the scalpel or in the demonstration of the bedside manner, receive occasionally a suitable honour; but this has so far been withheld from those who stand between pestilence and the people. If health is the most important asset for the individual and for the collection of individuals who make the State, it is surely not presumptuous to believe that those who save us from unnecessary sickness are worthy of some recognition.

WE live in days when Health Propaganda is rightly considered to be important. Without the sincere desire and co-operation of the nation as a whole, we are not likely to make much progress forward on the road to health. The distribution of a few knighthoods among some of our distinguished medical officers would do much to increase the prestige of their work in the minds of the people and would be excellent propaganda. As Shaw remarks in "Getting Married," "The whole strength of England lies in the fact that the enormous majority of the English people are snobs"; and, if that is the case, it is evident that they will regard the words of Sir Somebody Somebody with more reverence than they will give to those of plain Dr. So-and-So. The nation, seeing its health officers without honour, tends still to regard them as being merely experts in drain-testing and authorities on the flushing of a sewer; and this is an attitude of mind which every Honours List should seek to change.

WAILS about waste are many, but after making due allowance for our natural prejudice in favour of preventive medicine, we have no hesitation in placing in a class by itself a recent article in the "Pall Mall and Globe." The writer has his knife into most of the Ministries, but turns it with peculiar

**NOTES AND COMMENTS—(*cont.*).**

gusto in the vitals of the Ministry of Health. "What of its army of doctors and nurses and welfare assistants scattered over the country ? Are they giving value for their money ? " he asks. "*Is the service they are rendering so important that it cannot be dispensed with at a time like the present ?* What is the value of a school clinic to children whose fathers are out of work, because the clinic is using money which would otherwise be devoted to developing the trade in which they should be employed ? What is 'welfare' to the dinnerless, and 'town-planning' to the homeless ? These are the questions we have to ask and answer." If the sentence which we have italicised means anything at all, it suggests the closing down of the health services until better days shall arrive. If the writer has any acquaintance with the business world, he should know what is likely to be the fate of any elaborate organisation which, having ceased to function for a time, tries to go on again as though nothing had happened; but that is by the way. Money might certainly be saved by (for example) suspending the operation of the Food and Drugs Acts and removing the checks imposed on the sale of tuberculous meat and mixtures of milk and typhoid bacilli; by ceasing to collect house refuse; by permitting diphtheria contacts to circulate at their own sweet will. The sad case of a child underfed because its father is out of work is demonstrably improved by the slackening of the safeguards which keep it moderately clean and free from disease. Whether the burden of taxation and the amount of unemployment would be less in a period of assorted epidemics is another of those questions which we might ask and answer.

IN an interesting article upon the Economic Aspect of a Central Purchasing Organisation for Hospital Boards, the "Hospital World," of Toronto, mentions the opposition of private traders, which would seem excellent evidence of the saving that can be made by co-operative purchase. The writer quotes the success of the Australian Hospitals' Central Board of Supplies, but, in the main, devotes himself to the defending of the principle of a central purchasing board. He argues this so relentlessly that it is safe to assume an interested opposition from local retailers. On the other hand he does not deal with the position of those large boards whose orders are sufficiently big to enable them to buy direct from the manufacturers. He only prays them to "act unselfishly," and to "throw their orders in with the others so as to help their less fortunate neighbours." But, over here, one of the objections to co-operative purchase has been that the smaller or less well-managed institutions would benefit chiefly by it. We incline to believe that this argument applies only to group buying, but that, where central purchase exists on a very large scale, large and small hospitals may equally benefit. The experience of the British Red Cross Society during the war, when it supplied countless numbers of hospitals, points in this direction. Tentative attempts may very likely fail, but, in the end, we think that combination, here as elsewhere, is bound to secure economy.

"NO charity" is an amusing motto for a hospital, but the Manor House Orthopædic Hospital at Golders Green, has been credited with it. Our readers may recall that this institution is welcomed in Labour circles as the first Labour Hospital, and in itself it has several interesting features. According to "Burdett's Hospitals and Charities," it possesses 131 beds, and has an arrangement with the insurance companies whereby they may send the victims of industrial accidents for treatment at a capitation fee of twelve shillings a day. Established in 1916, the outcome of the Allies Benevolent Society, which, after the Truce, developed into the Industrial Orthopædic Society, the hospital's accounts show a substantial credit balance. The main source of income appears to be that of weekly contributions from the factories and shipyards, and the committee is the product of district councils, representatives of which form the governing body. We understand that each ward is represented on a committee of patients which advises the house committee and presumably helps to adjust complaints.

MEDICAL Officers of Health throughout the country will welcome the recent Circular (No. 269) from the Ministry of Health which frees them at last from the yearly penance of preparing a complicated "Annual Report." In future, at intervals of not more than five years, a "Survey Report" of a comprehensive nature will be required from each health area ; but in the intervening years an "Ordinary Report" will be sufficient. This ordinary report will be of a simple nature, and will take only a few days for its preparation. Perhaps later the Board of Education will follow the lead of the Ministry, and reduce the number and complexity of the tables of figures required from the school medical officers. Even in towns where the most careful system of record keeping is practised the compilation of these school medical service tables is a severe strain upon the ingenuity of the expert statistician.

DEATHS under anæsthetics continue to occur. At a recent inquest held by the City Coroner, Dr. F. J. Waldo, into a death that took place under an anæsthetic at Guy's Hospital, the Coroner is reported to have stated that in nearly all the deaths under anæsthetics that had been inquired into by him during the past twenty years, chloroform alone or in a mixture was the anæsthetic that was given. Fewer such deaths had come to his notice of late years, owing to the fact that the use of ether given by the open method was replacing that of chloroform. The jury in this case, in returning their verdict of death by misadventure, expressed the opinion that one qualified medical man should act as anæsthetist and that he should see the case through and not leave his patient under the influence of an anæsthetic in the charge of any other medical man. To apply the rota system in the administration of an anæsthetic seems to us not only ridiculous but of actual danger to the patient. The jury also considered that resident expert anæsthetists should be appointed at this Hospital, and they requested Dr. Waldo to forward their rider to the Guy's Hospital authorities.

